# Chronic Kidney Disease Among Agricultural Workers in Taiwan: A Nationwide Population-Based Study

**DOI:** 10.1016/j.ekir.2023.09.004

**Published:** 2023-09-09

**Authors:** Che-Jui Chang, Hsiao-Yu Yang

**Affiliations:** 1 Institute of Occupational and Environmental Health Sciences, National Taiwan University College of Public Health, Taipei, Taiwan; 2 Department of Family Medicine, National Taiwan University Hospital Hsin-Chu Branch, Hsinchu, Taiwan; 3 Department of Public Health, National Taiwan University College of Public Health, Taipei, Taiwan; 4 Department of Environmental and Occupational Medicine, National Taiwan University Hospital, Taipei, Taiwan; 5 Population Health and Welfare Research Center, National Taiwan University College of Public Health, Taipei, Taiwan

**Keywords:** chronic kidney disease of undetermined etiology, East Asia, farmers, health administrative data, heat stress, meteorological data

## Abstract

**Introduction:**

Chronic kidney disease (CKD) in agricultural communities is a significant public health issue. We aimed to investigate the epidemiology of CKD among Taiwanese farmers and its association with outdoor heat exposure.

**Methods:**

A nested case-control study was conducted on participants in the National Adult Health Examination (NAHE) from 2012 to 2018. The farming occupation was identified through National Health Insurance data. The primary outcomes of interest were the development of CKD, defined as a decreased estimated glomerular filtration rate (eGFR) with diagnosis by physicians, and CKD of undetermined etiology (CKDu), defined as CKD excluding common traditional etiologies. We calculated the county-wide average ambient temperature from a climate reanalysis dataset (ERA5-Land). All CKD cases were matched 1:2 to non-CKD participants by age and biological sex. We estimated the odds ratios (ORs) of CKD and CKDu for farmers and changes in mean ambient temperature (°C) before the examination.

**Results:**

We identified 844,412 farmers and 3,750,273 nonfarmers. Among 24.9% of farmers and 7.4% of nonfarmers with reduced kidney function, only 1 in 7 received a diagnosis of CKD. The farming occupation was independently predictive of CKDu (OR = 1.09, 95% confidence interval [CI] = 1.001–1.18) but not CKD. Increased ambient temperature (°C) was associated with a higher risk of CKD (OR = 1.023, 95% CI = 1.017–1.029), with particularly strong associations observed among middle-aged participants and diabetics.

**Conclusions:**

Taiwanese farmers might have a higher risk of developing CKDu. Outdoor heat exposure is associated with the development of CKD, and middle-aged participants and those with diabetes are more vulnerable than the general population.

## Graphical abstract

**Figure:**
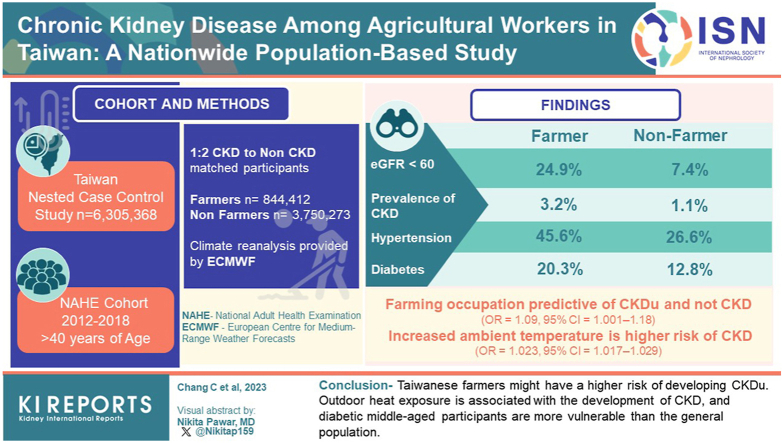

CKD is a significant public health issue worldwide, with traditional etiologies such as hypertension and diabetes being widely recognized. However, there is growing concern about a specific form of CKD known as CKDu or of nontraditional etiology, which has been predominantly reported in agricultural communities.^1^ CKDu is characterized by its association with heat stress, contaminated groundwater, and agrochemical use.^2^ The hotspots of CKDu have mostly been reported in agricultural communities in Central America, and South Asia, and in suspect locations such as Africa, the Middle East, and North America.^3^

The occupational risk factors for CKD vary across different regions. In recent years, the majority of CKDu cases found in Latin America have been young male farmers working under hot weather.^4^ Studies of CKDu in South Asia, such as Sri Lanka and India, have been focused on areas affected by contaminated groundwater.^2^ From observational studies in Korea and Japan, night shift workers have been found to have a higher risk of CKD.^5^^,^^6^ However, there is no consensus on the occupational risk factors for CKD among studies across different regions. In East Asia, which has a similar climatic geography to Latin America and a large agricultural population, epidemiologic data related to CKDu are less frequently reported.

As part of East Asia, Taiwan has a subtropical climate with hot and humid summers and mild winters. The average daily temperature typically reaches 30 °C during the rice-growing seasons in Taiwan. Because agricultural works are usually categorized as moderate-to-heavy workloads,^7^ the action limit for unacclimatized farmers and the threshold limit value for heat-acclimatized farmers for continuous work are below 25 °C and 28 °C, respectively.^8^ As a result, Taiwanese farmers may experience significant heat strain. With a farming population of 2.8 million, Taiwan's agricultural communities may be susceptible to an endemic prevalence of CKD, underscoring the need for heightened attention and awareness in these areas.^9^

Our previous retrospective study using pooled cross-sectional health screening data in Changhua County, Taiwan, had shown that the farmers had a 44% increased risk of CKDu compared to the nonfarmers.^10^ However, the population representativeness of that study was limited. Therefore, a nationwide population-based study is warranted to provide comprehensive epidemiological data on CKD among Taiwanese farmers.

In this study, we aim to contribute to the understanding of CKD among agricultural workers in Taiwan. While focusing on CKD, we also investigate the presence of CKDu as a secondary aspect. By investigating the risk of CKD/CKDu in Taiwanese farmers and its potential association with outdoor heat exposure, we hope to shed light on the epidemiology of CKD and CKDu in East Asia and provide valuable insights for preventive strategies and occupational health interventions.

## Methods

We conducted a nested case-control study on a cohort comprising participants of the NAHE from 2012 to 2018. NAHE, reimbursed by Taiwan’s Health Promotion Administration, provides nationwide free preventive health examination for beneficiaries of the National Health Insurance (99.6% population coverage) aged 40 years or older in general (detailed criteria in Supplementary Table S1). It includes the following items of examination:

1. Basic information: questionnaire (medical history, family history, medication history, health behaviors, depression screening, etc.)
2. Physical examination: general physical examination, height, weight, blood pressure, body mass index (BMI), waist circumference
3. Laboratory tests:
  (i) urine test: urine protein (qualitative or quantitative)
  (ii) eGFR calculation
  (iii) blood biochemical tests: aspartate aminotransferase, alanine aminotransferase, creatinine, blood glucose, blood lipids (total cholesterol, triglycerides, high-density lipoprotein cholesterol, low-density lipoprotein cholesterol calculation).
4. Hepatitis B surface antigen and hepatitis C antibody (detailed conditions in Supplementary Table S1).

The frequency of the service is in general once every 3 years for people aged 40–64 years, and once every year for indigenous individuals aged 55 years and above, individuals with poliomyelitis aged 35 years and above, and individuals aged 65 years and above. According to government statistics, approximately 30% of the eligible population in Taiwan received the service of NAHE.^11^ We acquired a dataset of NAHE from the Health and Welfare Data Science Center, Ministry of Health and Welfare of Taiwan. In addition, we acquired related datasets from the Health and Welfare Data Science Center to assess the demographic information and medical records of the participants.

All participants of NAHE aged 40 years or older in our data collection period (2012–2018) were potentially eligible for the study. If a participant attended NAHE multiple times, only their first visit was included. The exclusion criteria were as follows:

1. Participants whose health insurance was dependent on another family member: unable to determine whether their occupation was a farmer or not.
2. Death before 2013: potential follow-up period was too short.
3. Participants who did not attend the laboratory test.

The primary exposure of interest was the farming occupation (farmers vs. nonfarmers). In Taiwan, farmers participate in "Farmer's Health Insurance", which offers superior terms (e.g., lower insurance fees) as a special type of National Health Insurance. Therefore, participants' identities as farmers could be assessed from our datasets.

The primary outcome of interest was the development of CKD. The Kidney Disease Improving Global Outcomes collaboration has provided widely-used clinical criteria for CKD, which basically requires 2 measurements of impaired kidney function (eGFR <60 ml/min per 1.73 m^2^) based on serum creatinine. However, participants of NAHE only had 1 laboratory test; therefore, we defined a case of CKD as having an impaired kidney function at NAHE with subsequent diagnosis of CKD made by physicians during ≥2 outpatient visits in 1 year or 1 hospitalization. The subsequent CKD diagnoses by physicians allow for a distinction from minor acute kidney injury episodes. The Chronic Kidney Disease Epidemiology Collaboration formula was adopted to calculate the eGFR.^12^ We further applied the following 2 definitions of CKDu:

1. CKD of undetermined etiology - definition 1 (CKDu_1): a case of CKD with the exclusion of hypertension and diabetes.
2. CKD of undetermined etiology - definition 2 (CKDu_2): a case of CKD with the exclusion of hypertension, diabetes, glomerular diseases, congenital urinary diseases, and proteinuria at health examination (dipstick 1+, 30mg/dl or above). It is important to note that the prevalence of exercise-induced proteinuria might be higher because the health examinations do not require a morning fasting urine sample.

These definitions were modified from the Disadvantaged Populations eGFR Epidemiology Study (DEGREE) protocol,^13^ the International Society of Nephrology’s recommendations for population-based detection of CKDu,^14^ as well as the Mesoamerican and Sri Lankan definitions of suspected CKDu,^15^^,^^16^ but we removed the age requirements (age <60 or 70 years) because the mean age of Taiwanese farmers was 68 years old.^17^ Relevant diagnostic codes, except for proteinuria (assessed from health examination results), are summarized in the supplementary materials (Supplementary Table S2).

Other covariates collected from NAHE participants included demographic factors (age, sex, and residential region), lifestyle factors (alcohol drinking, betel nut chewing, and cigarette use), BMI, and comorbidities (hypertension, diabetes, hyperlipidemia, heart disease, chronic liver disease, gout/hyperuricemia, and urolithiasis). The identification of comorbidities was based on diagnostic codes made during medical care (Supplementary Table S2).

### Variables From Meteorological Data

From ERA5-Land, a climate reanalysis dataset provided by the European Centre for Medium-Range Weather Forecasts, we extracted hourly ambient temperature in Taiwan from 2011 to 2018.^18^ We converted these data into daily mean temperature using arithmetic mean and applied zonal estimation to aggregate the raster data and calculate the mean values within each geographic polygon. It allowed us to obtain historical representative temperatures for each county and provincial city. Therefore, for any given time point, we calculated a county-wide average of ambient temperature and linked it to the participants based on their time and place of health examination.

### Statistical Analysis

Under a nested case-control design, all CKD cases were individually matched 1:2 to non-CKD participants based on age (coarsened into groups of 3 years) and sex. The matching process was only conducted on matching CKD cases with non-CKD participants. When analyzing for CKDu, we excluded the CKD cases that did not meet the CKDu definition and excluded their matched controls (non-CKD participants), while retaining the CKDu cases along with their originally matched controls.

For descriptive analysis, we summarized the characteristics of the study population before and after case-control matching, including age (grouped by ten), gender, residential region, lifestyle factors, BMI, serum creatinine, eGFR, and comorbidities. Scalar variables were presented as means and SDs, whereas categorical variables were presented as numbers of observations and percentages.

For multivariable analysis, we performed conditional logistic regressions on the matched cohort to estimate the ORs of CKD and CKDu. The primary explanatory variable was the occupation (farmers vs. non-farmers). Other covariates included age (as a continuous variable in years), gender, residential region, lifestyle factors, BMI (normal, overweight, or underweight), and comorbidities. Missing data were summarized in the descriptive analysis but were excluded from the regression models. All statistical analyses were conducted using SAS software (V.9.4; SAS Institute), with a 2-tailed *P*-value of 0.05 considered statistically significant.

### Methods for Additional Analysis

To explore the temporal relationship between CKD risk and preceding environmental heat exposure, we used multiple logistic regression models to estimate the ORs of CKD for a 1-unit increase in average ambient temperature (°C) of different time-lagged structures. The primary explanatory variable was the average ambient temperature exposed over various lagged time frames, including the first to twelfth month before health examination (i.e., single lag structures) and within 1 to 12 months before health examination (i.e., cumulative lag structures). The regression analysis was iterated over each time frame of average ambient temperature. This method has been applied in previous literature for evaluating the time-lagged effect of different exposures.^19^, ^20^, ^21^

The same covariates used in the above-mentioned conditional logistic regressions were used in these additional analyses. Subgroup analyses were conducted based on age (over or under 65 years), sex, occupation (farmers and non-farmers), and disease status of hypertension and diabetes to identify potentially vulnerable populations to heat stress. We tested for differences of ORs within subgroups by assessing the significance of the interaction term between the subgrouping variable and the exposure (e.g., sex ∗ temperature) in the regression models. If an interaction reached significance (*P* for interaction < 0.05), then we could determine that the ORs differed significantly within the subgroups.

We estimated outdoor heat exposure using daily mean temperature by default and conducted sensitivity analyses using daily maximum and minimum temperatures. To account for other factors that affect heat stress, such as humidity, wind, and sun exposure, we estimated the daily mean, maximum, and minimum of wet bulb globe temperature. Wet bulb globe temperature was derived using an R package implementation (https://github.com/anacv/HeatStress) of the Liljegren formula.^22^

### Reporting Guidelines

The present study was conducted following the STROBE (Strengthening the Reporting of Observational Studies in Epidemiology) guidelines to ensure transparent and comprehensive reporting of the observational study.^23^

## Results

**Figure 1:**
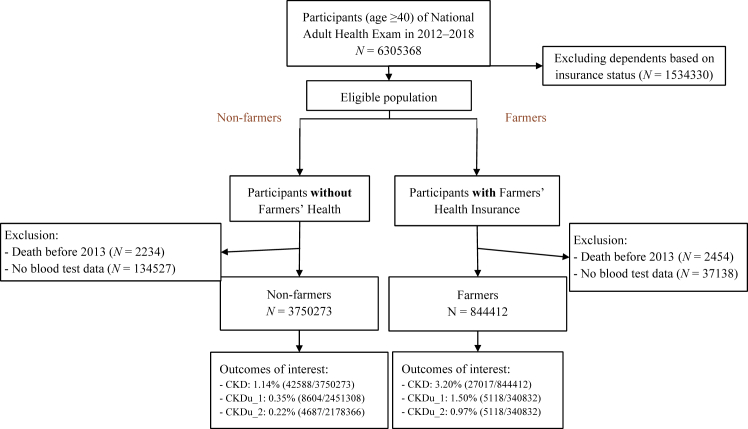
Study flowchart. The diagram outlines the steps of inclusion and exclusion of participants in this cohort study. Case definition of chronic kidney disease (CKD): an estimated glomerular filtration rate <60 ml/min per 1.73 m^2^ with subsequent diagnosis of CKD made by physicians during ≥2 outpatient visits in 1 year or 1 hospitalization. Chronic kidney disease of undetermined etiology - definition 1 (CKDu_1): a case of CKD with the exclusion of hypertension and diabetes. Chronic kidney disease of undetermined etiology - definition 2 (CKDu_2): a case of CKD with the exclusion of hypertension, diabetes, glomerular diseases, congenital urinary diseases, and proteinuria at health examination (dipstick 1+, 30 mg/dl or above).

Among 6,305,368 participants of NAHE in 2012–2018, we identified 844,412 farmers and 3,750,273 non-farmers through prespecified inclusion/exclusion criteria. Detailed numbers of participants at each step of data collection were shown in a flow diagram (Figure 1).

Compared with other participants, farmers were on average significantly older (67.3 vs. 54.0 years old), more likely to live in Central and Southern Taiwan (78.4% vs. 53.2%) where farmland prevails, more likely to have hypertension (45.6% vs. 26.0%), diabetes (20.3% vs. 12.8%), and heart diseases (10.2% vs. 5.0%). Table 1 summarizes the characteristics of the study population. Among farmers and non-farmers with reduced kidney function (eGFR <60, 24.9% vs. 7.4%), only a few were subsequently diagnosed with CKD (prevalence: 3.2% vs. 1.1%), and fewer met our prespecified criteria of CKDu (prevalence of CKDu_1: 1.5% vs. 0.4%; prevalence of CKDu_2: 1.0% vs. 0.2%) (Figure 1).

**Table 1:** Characteristics of the study population

Characteristics	Farmers (*N* = 844,412)	Nonfarmers (*N* = 3,750,273)	*P*-value
Age (yr)			
Mean	67.27 (12.24)	54.04 (10.44)	<0.001
40–49	83,253 (9.9)	1,410,708 (37.6)	<0.001
50–59	145,914 (17.3)	1,379,521 (36.8)	
60–69	213,751 (25.3)	660,065 (17.6)	
70–79	261,939 (31.0)	179,912 (4.8)	
Over 80	139,555 (16.5)	120,067 (3.2)	
Sex			
Female	448,522 (53.1)	1,983,745 (52.9)	<0.001
Male	395,890 (46.9)	1,766,528 (47.1)	
Region			
Northern	143,757 (17.0)	1,631,453 (43.5)	<0.001
Central	335,683 (39.8)	878,203 (23.4)	
Southern	325,637 (38.6)	1,118,758 (29.8)	
Eastern	32,945 (3.9)	93,082 (2.5)	
Outlying islands	6390 (0.8)	28,777 (0.8)	
Lifestyle			
Cigarette use	88,768 (10.6)	584,411 (15.6)	<0.001
Alcohol drinking	20,843 (2.5)	127,185 (3.4)	<0.001
Betel nut chewing	41,065 (4.9)	183,629 (4.9)	0.201
Exercise	362,963 (43.5)	1,636,137 (44.3)	<0.001
Physique (BMI)			
Normal (18.5–24)	334,054 (41.2)	1,631,302 (45.0)	<0.001
Under (<18.5)	26,559 (3.3)	112,005 (3.1)	
Over (≥24)	449,851 (55.5)	1,886,037 (52.0)	
Renal profiles^a^			
CRE (mg/dl)	1.07 (1.88)	0.96 (1.94)	<0.001
eGFR (ml/min per 1.73 m^2^)	72.96 (21.25)	86.80 (19.22)	<0.001
eGFR 30–59	185,306 (21.9)	244,540 (6.5)	<0.001
eGFR 15–29	15,783 (1.9)	17,248 (0.5)	
eGFR <15	7775 (0.9)	15,630 (0.4)	
Comorbidities			
Hypertension	384,682 (45.6)	974,623 (26.0)	<0.001
Diabetes	170,955 (20.3)	480,792 (12.8)	<0.001
Hyperlipidemia	176,197 (20.9)	685,088 (18.3)	<0.001
Heart disease	86,347 (10.2)	187,642 (5.0)	<0.001
Chronic liver disease	7372 (0.9)	57,811 (1.5)	<0.001
Gout / hyperuricemia	58,216 (6.9)	186,995 (5.0)	<0.001
Urolithiasis	34,369 (4.1)	155,459 (4.2)	<0.001

BMI, body mass index; CKD, chronic kidney disease; CKD-EPI, Chronic Kidney Disease Epidemiology Collaboration; CRE, serum creatinine; eGFR, estimated glomerular filtration rate.

The study population comprised participants of National Adult Health Examination in 2012–2018.

The SD is presented in parentheses, following the mean for scalar variables. The percentage is presented in parentheses following the count for categorical variables.

The hypothesis testing for differences between groups: scalar variables were analyzed using the Student's t-test, whereas dichotomous variables were assessed using the Chi-squared test. Multi-categorical variables were evaluated using the Chi-squared test of independence. The significance level was set at *P* < 0.05.

Missing data (as a percentage of observations): region, 11.5%; cigarette use, 0.3%; alcohol drinking, 0.3%; betel nut chewing, 0.3%; exercise, 1.5%; BMI, 3.4%; renal profile, 1.1%.

a The CKD-EPI formula was adopted to calculate the eGFR (as the renal function).

Before multivariable analysis, all 69,605 participants with CKD (cases) were matched to the non-CKD counterparts (controls) in a 1:2 ratio based on age and sex. Compared to the matched control group, cases were more likely to live in Central and Southern Taiwan, and have more comorbidities such as hypertension, diabetes, hyperlipidemia, heart diseases, gout/hyperuricemia, and urolithiasis (Table 2).

**Table 2:** Characteristics of matched study population: CKD cases and non-CKD participants

Characteristics	CKD cases (*N* = 69,605)	Non-CKD (*N* =139,210)	*P*-value
Age (yr)			
Mean	70.64 (12.45)	70.59 (12.46)	0.387
40–49	4041 (5.8)	8082 (5.8)	0.877
50–59	10,182 (14.6)	20,603 (14.8)	
60–69	16,438 (23.6)	32,793 (23.6)	
70–79	19,282 (27.7)	38,408 (27.6)	
Over 80	19,662 (28.3)	39,324 (28.3)	
Sex			
Female	24,397 (35.1)	48,794 (35.1)	1.000
Male	45,208 (65.0)	90,416 (65.0)	
Region			
Northern	19,869 (28.6)	49,128 (35.3)	<0.001
Central	20,914 (30.1)	39,089 (28.1)	
Southern	25,782 (37.0)	44,913 (32.3)	
Eastern	2621 (3.8)	4733 (3.4)	
Outlying islands	419 (0.6)	1347 (1.0)	
Occupation			
Farmers	27,017 (38.8)	54,485 (39.1)	0.154
Non-farmers	42,588 (61.2)	84,725 (60.9)	
Lifestyle			
Cigarette use	7418 (10.7)	17,265 (12.4)	<0.001
Alcohol drinking	1160 (1.7)	3908 (2.8)	<0.001
Betal nut chewing	2357 (3.4)	5178 (3.7)	<0.001
Exercise	27,543 (40.3)	63,680 (46.5)	<0.001
Physique (BMI)			
Under (<18.5)	2621 (4.0)	5709 (4.3)	<0.001
Over (≥24)	37,766 (57.0)	70,018 (52.4)	
Normal (18.5–24)	25,891 (39.1)	57,839 (43.3)	
Comorbidities			
Hypertension	47,821 (68.7)	66,080 (47.5)	<0.001
Diabetes	28,389 (40.8)	28,519 (20.5)	<0.001
Hyperlipidemia	23,033 (33.1)	30,006 (21.6)	<0.001
Heart disease	16,315 (23.4)	16,894 (12.1)	<0.001
Chronic liver disease	1117 (1.6)	1419 (1.0)	<0.001
Gout / hyperuricemia	15,872 (22.8)	10,100 (7.3)	<0.001
Urolithiasis	5495 (7.9)	5818 (4.2)	<0.001

BMI, body mass index; CKD, chronic kidney disease; eGFR, estimated glomerular filtration rate.

Participants with CKD were identified by an eGFR <60 ml/min per 1.73 m^2^ and subsequent diagnosis of CKD made by physicians. Coarsened exact matching was performed to facilitate further multivariable analysis (Table 3). All CKD cases were matched 1:2 with non-CKD participants, based on age (coarsened into groups of 3 years) and sex.

The SD is presented in parentheses following the mean for scalar variables. The percentage is presented in parentheses following the count for categorical variables.

The hypothesis testing for differences between groups: Scalar variables were analyzed using the Student's t-test, whereas dichotomous variables were assessed using the Chi-squared test. Multi-categorical variables were evaluated using the Chi-squared test of independence. The significance level was set at *P* < 0.05.

In Table 3, we summarize the multivariable analysis for factors associated with CKD and CKDu. Occupation as farmers was inconsistently associated with CKDu (CKDu_1: OR = 1.09, 95% CI = 1.001–1.18; CKDu_2: OR = 1.09, 95% CI = 0.98–1.22), and no significant association was found with farming occupation and CKD. Living in the less urbanized region (Central, Southern, and Eastern Taiwan), being underweight (BMI <18.5), and having more comorbid conditions were associated with both CKD and CKDu. Conversely, being either overweight, having habits of smoking, alcohol drinking, and exercising were negatively associated with both CKD and CKDu.

**Table 3:** Analysis of the variables associated with CKD and CKDu

Covariate	CKD		CKDu_1		CKDu_2	
	Crude OR	Adjusted OR	Crude OR	Adjusted OR	Crude OR	Adjusted OR
Farmers	0.99 (0.97–1.00) *P =* 0.197	0.90 (0.87–0.94)^a^ *P <* 0.001	1.14 (1.09–1.19)^b^ *P <* 0.001	1.09 (1.00–1.18)^b^ *P =* 0.041	1.15 (1.09–1.22)^b^ *P <* 0.001	1.09 (0.98–1.22) *P =* 0.123
Region						
Northern	1.00	1.00	1.00	1.00	1.00	1.00
Central	1.31 (1.28–1.35)^b^ *P <* 0.001	1.73 (1.39–1.48)^b^ *P <* 0.001	1.47 (1.40–1.56)^b^ *P <* 0.001	1.51 (1.42–1.61)^b^ *P <* 0.001	1.56 (1.44–1.68)^b^ *P <* 0.001	1.58 (1.45–1.73)^b^ *P <* 0.001
Southern	1.43 (1.40–1.47)^b^ *P <* 0.001	1.51 (1.47–1.55)^b^ *P <* 0.001	1.54 (1.46–1.62)^b^ *P <* 0.001	1.56 (1.47–1.65)^b^ *P <* 0.001	1.59 (1.48–1.71)^b^ *P <* 0.001	1.63 (1.51–1.77)^b^ *P <* 0.001
Eastern	1.39 (1.31–1.46)^b^ *P <* 0.001	1.37 (1.29–1.46)^b^ *P <* 0.001	1.21 (1.07–1.38)^b^ *P =* 0.003	1.18 (1.03–1.36)^b^ *P =* 0.019	1.34 (1.14–1.58)^b^ *P <* 0.001	1.34 (1.12–1.61)^b^ *P =* 0.002
Outlying islands	0.84 (0.76–0.94)^a^ *P =* 0.001	0.74 (0.65–0.83)^a^ *P <* 0.001	0.57 (0.43–0.76)^a^ *P <* 0.001	0.54 (0.40–0.72)^a^ *P <* 0.001	0.74 (0.52–1.03) *P =* 0.084	0.62 (0.42–0.89)^a^ *P =* 0.013
Lifestyle						
Cigarette use	0.84 (0.82–0.87)^a^ *P <* 0.001	0.90 (0.86–0.93)^a^ *P <* 0.001	0.79 (0.74–0.84)^a^ *P <* 0.001	0.82 (0.75–0.89)^a^ *P <* 0.001	0.78 (0.71–0.85)^a^ *P <* 0.001	0.81 (0.73–0.91)^a^ *P <* 0.001
Alcohol drinking	0.59 (0.55–0.63)^a^ *P <* 0.001	0.59 (0.54–0.64)^a^ *P <* 0.001	0.57 (0.50–0.66)^a^ *P <* 0.001	0.63 (0.53–0.75)^a^ *P <* 0.001	0.59 (0.48–0.71)^a^ *P <* 0.001	0.59 (0.47–0.75)^a^ *P <* 0.001
Betal nut chewing	0.91 (0.86–0.95)^a^ *P <* 0.001	0.93 (0.87–1.00)^a^ *P =* 0.041	0.82 (0.73–0.92)^a^ *P <* 0.001	0.96 (0.83–1.10) *P =* 0.582	0.83 (0.71–0.96)^a^ *P =* 0.015	0.99 (0.82–1.20) *P =* 0.924
Exercise	0.78 (0.76–0.79)^a^ *P <* 0.001	0.80 (0.79–0.82)^a^ *P <* 0.001	0.82 (0.78–0.85)^a^ *P <* 0.001	0.83 (0.79–0.87)^a^ *P <* 0.001	0.87 (0.82–0.91)^a^ *P <* 0.001	0.86 (0.80–0.91)^a^ *P <* 0.001
Physique (BMI)						
Under (<18.5)	1.02 (0.98–1.07) *P = 0.384*	1.17 (1.11–1.24) *P <* 0.001	1.42 (1.30–1.56) *P <* 0.001	1.44 (1.29–1.60) *P <* 0.001	1.27 (1.12–1.44) *P <* 0.001	1.28 (1.11–1.48) *P <* 0.001
Over (≥24)	1.20 (1.18–1.23) *P <* 0.001	0.93 (0.91–0.95)^a^ *P <* 0.001	0.77 (0.73–0.80)^a^ *P <* 0.001	0.68 (0.65–0.72)^a^ *P <* 0.001	0.78 (0.74–0.83)^a^ *P <* 0.001	0.70 (0.65–0.74)^a^ *P <* 0.001
Normal (18.5–24)	1	1	1	1	1	1
Comorbidities						
Hypertension	2.43 (2.38–2.48)^b^ *P <* 0.001	1.98 (1.94–2.03)^b^ *P <* 0.001	–	–	–	–
Diabetes	2.67 (2.62–2.73)^b^ *P <* 0.001	2.30 (2.25–2.36)^b^ *P <* 0.001	–	–	–	–
Hyperlipidemia	1.80 (1.77–1.84)^b^ *P <* 0.001	1.28 (1.25–1.31)^b^ *P <* 0.001	1.17 (1.11–1.23)^b^ *P <* 0.001	1.17 (1.10–1.24)^b^ *P <* 0.001	1.37 (1.28–1.46)^b^ *P <* 0.001	1.37 (1.27–1.48)^b^ *P <* 0.001
Heart disease	2.21 (2.16–2.26)^b^ *P <* 0.001	2.00 (1.94–2.06)^b^ *P <* 0.001	1.81 (1.71–1.91)^b^ *P <* 0.001	1.89 (1.77–2.02)^b^ *P <* 0.001	1.71 (1.59–1.84)^b^ *P <* 0.001	1.76 (1.61–1.92)^b^ *P <* 0.001
Chronic liver disease	1.59 (1.47–1.72)^b^ *P <* 0.001	1.48 (1.35–1.63)^b^ *P <* 0.001	1.77 (1.50–2.08)^b^ *P <* 0.001	1.71 (1.42–2.06)^b^ *P <* 0.001	1.77 (1.43–2.20)^b^ *P <* 0.001	1.65 (1.28–2.12)^b^ *P <* 0.001
Gout / hyperuricemia	3.78 (3.68–3.88)^b^ *P <* 0.001	3.75 (3.63–3.87)^b^ *P <* 0.001	3.92 (3.69–4.17)^b^ *P <* 0.001	4.27 (3.98–4.58)^b^ *P <* 0.001	4.25 (3.92–4.61)^b^ *P <* 0.001	4.59 (4.17–5.04)^b^ *P <* 0.001
Urolithiasis	1.97 (1.89–2.04)^b^ *P <* 0.001	1.82 (1.74–1.90)^b^ *P <* 0.001	2.34 (2.15–2.54)^b^ *P <* 0.001	2.24 (2.04–2.46)^b^ *P <* 0.001	2.35 (2.11–2.63)^b^ *P <* 0.001	2.33 (2.05–2.65)^b^ *P <* 0.001

The odds ratios (OR) of CKD and CKDu were estimated by logistic regression. Age and sex were adjusted by case-control matching (Table 2) as well as the regression model. The other covariates adjusted for the analysis included occupation, residential region, lifestyle factors, BMI, and comorbidities. The 95% confidence intervals are presented in parentheses following the risk estimates.

Case definition of chronic kidney disease (CKD): an estimated glomerular filtration rate < 60 ml/min/1.73 m^2^ with subsequent diagnosis of CKD made by physicians during ≥2 outpatient visits in one year or one hospitalization.

Chronic kidney disease of undetermined etiology - definition 1 (CKDu_1): a case of CKD with the exclusion of hypertension and diabetes.

Chronic kidney disease of undetermined etiology - definition 2 (CKDu_2): a case of CKD with the exclusion of hypertension, diabetes, glomerular diseases, congenital urinary diseases, and proteinuria at health examination (dipstick 1+, 30 mg/dl or above).

a Negative association with statistical significance (*P* < 0.05).

b Positive association with statistical significance (*P* < 0.05).

### Additional Analysis

The association between CKD/CKDu and preceding outdoor heat exposure of various durations was explored in the matched cohort (Table 4). Our analysis investigated the effects of average ambient temperature on CKD using different lag structures, including single month lags and cumulative month lags. Notably, the cumulative exposure to increased ambient temperature within 12 months (referred to as lag 0–12) before the health examination demonstrated a significant association with a higher risk of CKD (OR = 1.023, 95% CI = 1.017–1.029). Several other cumulative lags (lag 0–1, lag 0–2, lag 0–9, lag 0–10, and lag 0–11) of outdoor heat exposure also exhibited statistically significant associations with CKD. These findings suggest that chronic exposure to increased ambient temperature may contribute to the development of CKD. On the other hand, the association between CKDu and cumulative exposure of increased ambient temperature was not significant.

**Table 4:** Associations between CKD and the average ambient temperature: multivariable analyses for various time lags

Lag structure	Adjusted odds ratio		
	CKD	CKDu_1	CKDu_2
Single lag: average ambient temperature of the *N*th month before the examination (°C)			
lag 1	1.005 (1.003–1.008)^a^ *P* < 0.001	1.003 (0.996–1.009) *P* = 0.371	1.000 (0.991–1.009) *P* = 1.000
lag 2	1.002 (0.999–1.004) *P* = 0.117	0.997 (0.990–1.003) *P* = 0.373	0.993 (0.985–1.002) *P* = 0.107
lag 3	0.998 (0.995–1.000) *P* = 0.117	0.990 (0.984–0.997)^b^ *P* = 0.003	0.988 (0.979–0.997)^b^ *P* = 0.009
lag 4	0.995 (0.993–0.998)^b^ *P* < 0.001	0.986 (0.980–0.993)^b^ *P* < 0.001	0.984 (0.975–0.993)^b^ *P* < 0.001
lag 5	0.995 (0.993–0.998)^b^ *P* < 0.001	0.986 (0.980–0.993)^b^ *P* < 0.001	0.985 (0.976–0.994)^b^ *P* = 0.001
lag 6	0.997 (0.995–1.000)^b^ *P* = 0.019	0.991 (0.984–0.997)^b^ *P* = 0.007	0.991 (0.982–1.000) *P* = 0.051
lag 7	1.002 (1.000–1.005) *P* = 0.116	1.001 (0.994–1.007) *P* = 0.776	1.001 (0.992–1.010) *P* = 0.839
lag 8	1.007 (1.004–1.009)^a^ *P* < 0.001	1.009 (1.002–1.016)^a^ *P* = 0.011	1.009 (1.000–1.018)^a^ *P* = 0.049
lag 9	1.012 (1.009–1.015)^a^ *P* < 0.001	1.018 (1.011–1.025)^a^ *P* < 0.001	1.018 (1.009–1.028)^a^ *P* < 0.001
lag 10	1.015 (1.012–1.018)^a^ *P* < 0.001	1.021 (1.014–1.028)^a^ *P* < 0.001	1.022 (1.012–1.031)^a^ *P* < 0.001
lag 11	1.014 (1.011–1.017)^a^ *P* < 0.001	1.017 (1.010–1.024)^a^ *P* < 0.001	1.017 (1.008–1.027)^a^ *P* < 0.001
lag 12	1.011 (1.008–1.013)^a^ *P* < 0.001	1.010 (1.003–1.016)^a^ *P* = 0.003	1.008 (0.999–1.017) *P* = 0.080
Cumulative lag: average ambient temperature within *N* months before the examination(°C)			
lag 0–1	1.005 (1.003–1.008)^a^ *P* < 0.001	1.003 (0.996–1.009) *P* = 0.371	1.000 (0.991–1.009) *P* = 1.000
lag 0–2	1.004 (1.001–1.006)^a^ *P* = 0.002	1.000 (0.993–1.007) *P* = 1.000	0.996 (0.987–1.005) *P* = 0.392
lag 0–3	1.002 (0.999–1.005) *P* = 0.192	0.996 (0.989–1.003) *P* = 0.267	0.992 (0.983–1.002) *P* = 0.100
lag 0–4	1.000 (0.997–1.003) *P* = 1.000	0.992 (0.984–0.999)^b^ *P* = 0.037	0.988 (0.978–0.998)^b^ *P* = 0.019
lag 0–5	0.998 (0.995–1.002) *P* = 0.266	0.987 (0.979–0.996)^b^ *P* = 0.003	0.983 (0.972–0.995)^b^ *P* = 0.004
lag 0–6	0.997 (0.994–1.001) *P* = 0.093	0.984 (0.975–0.993)^b^ *P* < 0.001	0.980 (0.967–0.993)^b^ *P* = 0.003
lag 0–7	0.998 (0.994–1.002) *P* = 0.333	0.983 (0.972–0.993)^b^ *P* = 0.002	0.979 (0.964–0.993)^b^ *P* = 0.005
lag 0–8	1.000 (0.996–1.005) *P* = 1.000	0.984 (0.973–0.996)^b^ *P* = 0.007	0.980 (0.964–0.996)^b^ *P* = 0.015
lag 0–9	1.005 (1.000–1.010)^a^ *P* = 0.049	0.990 (0.977–1.003) *P* = 0.134	0.985 (0.967–1.003) *P* = 0.105
lag 0–10	1.012 (1.006–1.017)^a^ *P* < 0.001	0.998 (0.984–1.013) *P* = 0.799	0.993 (0.973–1.013) *P* = 0.504
lag 0–11	1.019 (1.013–1.025)^a^ *P* < 0.001	1.006 (0.990–1.021) *P* = 0.456	1.000 (0.979–1.022) *P* = 1.000
lag 0–12	1.023 (1.017–1.029)^a^ *P* < 0.001	1.010 (0.994–1.026) *P* = 0.220	1.004 (0.983–1.026) *P* = 0.728

The table summarizes the odds ratios and 95% confidence intervals of CKD per unit increase in average ambient temperature (°C) for different lag structures, including the single month lags and the cumulative month lags. For example, lag 3 represents the average ambient temperature of the third month before the health examination, and lag 0–12 represents the average ambient temperature within 12 months before the examination. The model adjusted the odds ratios for age, sex, occupation, residential region, lifestyle factors, BMI, and comorbidities.

Case definition of chronic kidney disease (CKD): an estimated glomerular filtration rate < 60 ml/min/1.73 m^2^ with subsequent diagnosis of CKD made by physicians during ≥2 outpatient visits in one year or one hospitalization.

Chronic kidney disease of undetermined etiology - definition 1 (CKDu_1): a case of CKD with the exclusion of hypertension and diabetes.

Chronic kidney disease of undetermined etiology - definition 2 (CKDu_2): a case of CKD with the exclusion of hypertension, diabetes, glomerular diseases, congenital urinary diseases, and proteinuria at health examination (dipstick 1+, 30mg/dl or above).

a Positive association with statistical significance (*P* < 0.05).

b Negative association with statistical significance (*P* < 0.05).

**Figure 2:**
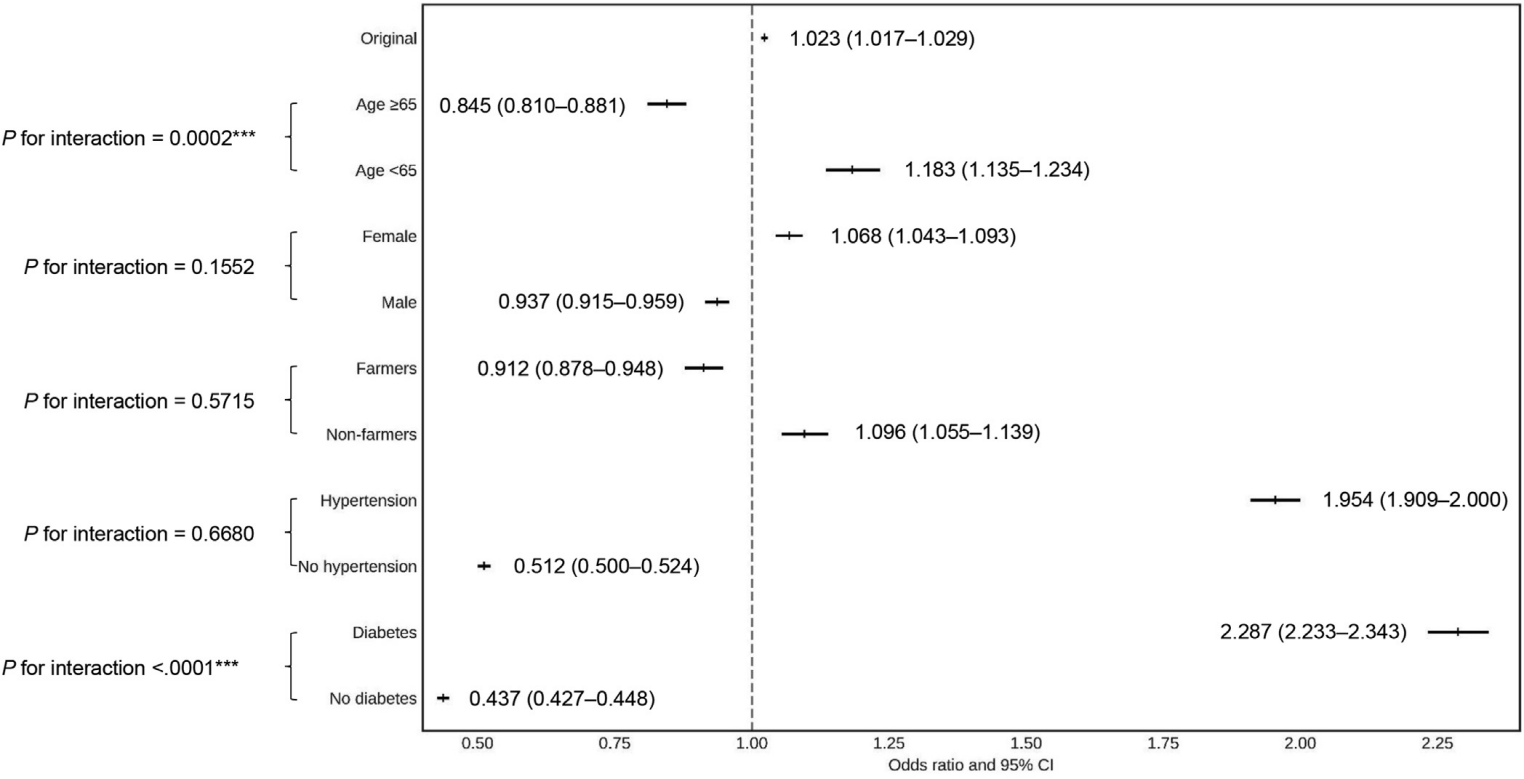
Subgroup analysis for the association between CKD and the average ambient temperature within 12 months before the examination. For the original study population and various subgroups, the error bar plot presents the odds ratios and corresponding 95% confidence intervals of chronic kidney disease (CKD) per unit increase in average ambient temperature (°C) within the 12 months preceding the examination. The odds ratios were adjusted for factors including age, sex, occupation, residential region, lifestyle factors, body mass index (BMI), and comorbidities. In the plot, the vertical bars represent the odds ratios, whereas the solid horizontal lines represent the corresponding 95% confidence intervals. The vertical dashed line represents an odds ratio of 1, indicating no increased risk. Therefore, any vertical bar with its horizontal line positioned entirely to the right of this dashed line indicates an elevated risk of CKD associated with increased temperature.

Because of the significant association between CKD and ambient temperature, we conducted additional subgroup analysis to further investigate the findings. (Figure 2; Supplementary Figure S1) In the subgroup analysis, age (*P* for interaction = 0.0002) and diabetes status (*P* for interaction < 0.0001) yielded significant interactions with the associations of interest, showing particularly strong associations between outdoor heat exposure and CKD among middle-aged participants (OR = 1.183, 95% CI = 1.135–1.234) and diabetics (OR = 2.287, 95% CI = 2.233–2.343).

In the sensitivity analysis, we examined the robustness of our results by replacing the average temperature with the daily maximum, daily minimum, or wet bulb globe temperature. The results remained consistent and were not significantly affected by these alternative temperature measures (Supplementary Table S3)

## Discussion

### Main Findings

The study examined the association between farming occupation and CKD. We found that farmers had a 9% higher risk of CKDu compared to nonfarmers, and this finding could potentially be applied to the farming population of 2.8 million in Taiwan. However, it is important to note that the association between farming occupation and CKDu was found to be inconsistent. In our analysis, we observed a significant association in a less strict definition of CKDu (CKDu_1: OR = 1.09, 95% CI = 1.001–1.18) which only excluded hypertension and diabetes, but not in the one (CKDu_2: OR = 1.09, 95% CI = 0.98–1.22) that further excluded proteinuria and diagnosis of glomerular diseases and congenital urinary diseases. Although the inconsistency might only be caused by lower case number in a stricter definition of CKDu, this suggests that the relationship between farming occupation and CKDu may vary depending on the specific measure used. Furthermore, we did not find a significant association between farming occupation and CKD. This observation may indicate that the increased risk of kidney disease among farmers primarily pertains to the normotensive nondiabetic CKD.

Further, we discovered that a large proportion of participants with reduced kidney function were undiagnosed with CKD. Among 24.9% of farmers and 7.4% of nonfarmers with eGFR <60 ml/min per 1.73 m^2^, only 1 in 7 received a diagnosis of CKD (prevalence of 3.2% and 1.1%, respectively). This indicates a significant underdiagnosis of CKD in both farming and nonfarming populations.

In addition, the study explored the association between outdoor heat exposure and kidney function decline. We found that increased outdoor heat exposure was associated with higher risks of CKD (2.3% per °C increase), indicating that heat exposure may contribute to the development of kidney disease. It is noteworthy that middle-aged participants and those with diabetes showed a particularly strong vulnerability to the effects of outdoor heat exposure on kidney function decline, highlighting the importance of considering these factors in preventive strategies.

### Comparing Risk Factors of CKDu in Taiwan and the World

Our finding that farming occupation potentially serves as a risk factor for CKDu, but not for overall CKD, is consistent with the worldwide epidemic of CKD in agricultural communities. This higher risk of CKD among agricultural workers, especially among young male farmers, is typically not linked to diabetes and hypertension.^24^^,^^25^ A recent meta-analysis by Chapman *et al.* included 4 systematic reviews and 61 primary studies on risk factors of CKDu.^26^ It also concluded that working in agriculture is the major risk factor for CKDu. However, other potential risk factors such as agrochemicals, heat stress, and heavy metals were not significant when pooling eligible studies worldwide. It is probable that the development of CKDu in agricultural communities is multifactorial and that the primary contributor varies across regions.

Other risk factors of CKD/CKDu in our study included old age, living in less urbanized regions (e.g., Central, Southern, and Eastern Taiwan), and having certain comorbidities. These are well-documented risk factors of CKD.^27^^,^^28^ Lifestyle factors such as BMI and exercise habits may not necessarily indicate a causal relationship with CKD/CKDu, because it is difficult to determine which factor occurred first. It is possible that after receiving a CKD diagnosis, patients may make lifestyle changes such as reducing alcohol and tobacco use and decreasing food intake, which could contribute to the observed associations. Other explanations may include the possibility of competing risks of fatal diseases (related to alcohol and tobacco use) or residual confounding. In addition, because the NAHE focuses on people without known chronic disease and that our datasets were pooled cross-sectional data, we could not determine the timing of new CKD diagnoses, particularly in relation to lifestyle factors, as a typical cohort study would. Ultimately, more research is needed to identify multifactorial risk factors for CKD/CKDu and the causal relationships, especially in agricultural communities.

Regarding the performance of Chronic Kidney Disease Epidemiology Collaboration in this population and the potential risk of overestimation of CKD, particularly in older individuals, it is noteworthy that the prevalence of CKD in older individuals in the US appears to be relatively high when comparing it to the incidence of treated kidney failure based on the Chronic Kidney Disease Epidemiology Collaboration and MDRD formulas. One possible explanation for this disparity is the existence of competing risks, particularly in relation to fatal cardiovascular diseases.^12^ However, considering the high prevalence and incidence of end-stage renal disease in Taiwan, as well as the elevated prevalence and limited awareness of CKD, we propose that maintaining an eGFR <60 as the cutoff for stage 3 CKD, in accordance with the Kidney Disease Improving Global Outcomes guidelines and Taiwan's CKD guidelines, is a reasonable approach.

### Special Considerations for Characteristics of Taiwanese Agriculture

The agriculture industry in Taiwan exhibits notable differences compared to agricultural practices in Mesoamerican countries, where CKDu epidemiology is well-studied. In Taiwan, large-scale contracted farming is not the predominant approach. Instead, a small-scale, family-based pattern of agricultural work prevails, with an average household size of 4.09 and 81.8% of farming households operating on farms of 1.0 hectares or smaller.^29^ The majority of agricultural workers in Taiwan are self-employed or family members, and they receive no formal occupational health education. According to the 2015 Taiwan Agricultural Census, the top 3 agricultural industries with the highest number of households (*N* = 998) are rice cultivation (60.0%), mixed grain cultivation (16.8%), and vegetable cultivation (12.0%).^29^ This unique context suggests that the heat exposure and CKD risk may differ from those observed in regions with different agricultural structures. In addition, it is important to note the influence of machinery on agricultural practices. The introduction of automated machinery, such as automatic fruit and vegetable harvesters, can alter the nature of work and its associated heat strain. Future research should further explore the occupational risks for agricultural workers cultivating different crops to better understand the heat-related risks among them.

### Outdoor Heat Exposure and CKD

In the additional analysis, we found an association between increased ambient temperature and increased risk of CKD (Table 4). Studies of the pathophysiology of heat stress-related CKD have identified several plausible mechanisms, including elevated renin-angiotensin-aldosterone system, renal sympathetic activity, rhabdomyolysis-induced hyperuricemia, and fructokinase-mediated hyperuricemia.^30^ Although most of the studies in Asia have focused on contaminated groundwater instead of heat stress compared to those in Latin America,^2^ the rice-growing seasons in Taiwan have daily mean temperatures as high as 30 °C, which is theoretically sufficient to cause heat stress-related injuries under a moderate-to-heavy workload.^8^

Older people are usually more susceptible to heat injuries than younger people under the same level of heat exposure, due to age-related alterations of sweating, skin blood flow, and cardiovascular function.^31^ However, our study found that increased ambient temperature was more associated with increased risk of CKD in middle-aged participants than in the elderly. In previous studies, CKD among agricultural communities was also found mainly in younger people.^1^^,^^4^ This may be because younger outdoor workers are more likely to be exposed to hot weather for long periods of time.

People with diabetes are at high risk of developing CKD, or diabetic kidney disease.^32^ Our study also found that increased ambient temperature was associated with an increased risk of CKD, particularly in diabetic patients. This may be due to microvasculopathy and macrovasculopathy, and impaired capacity to dissipate heat, making patients with diabetes less tolerant to heat stress.^33^^,^^34^

We expected the farmer subgroup to show a higher association with increased ambient temperature and CKD; however, the results were not significant. This may be because the nonfarmers subgroup also has many outdoor workers; therefore, the interaction between farming occupation and ambient temperature on the risk of CKD did not reach significance. Further, the occupational risks of the farmers are not only heat stress but also agrochemicals, heavy metal exposure, etc. Therefore, we cannot assume that the higher risk of CKDu among farmers can be fully explained by heat stress.

Recently the impact of air pollution, particularly particulate matter 2.5 absorbance (a proxy for elemental carbon), on human health has garnered significant attention, including its association with an increased risk of CKD.^35^^,^^36^ Although our analysis primarily focused on the CKD risk associated with outdoor heat exposure, it is crucial to consider the potential influence of air pollution, including its seasonal trends and coexposure with heat stress. Given the observed association between heat exposure and CKD in our study, investigating the interplay between air pollution and temperature could be a worthwhile path for future research.

### Low Diagnostic Rate of CKD

Although we found 24.9% of farmers and 7.4% of nonfarmers had reduced kidney function (eGFR <60 ml/min per 1.73 m^2^), the prevalence of CKD diagnosis was low in both farmers (3.2%) and nonfarmers (1.1%). Previous nationwide studies in Taiwan have shown that the prevalence of CKD stage 3 to 5 ranged from 6.9% to 7.1%.^37^^,^^38^ In a cross-sectional study of 2210 adults in Uddanam, India, the prevalence of CKDu was 13.3%.^39^ In another cross-sectional study of 354 male farmers in West Java, Indonesia, the prevalence of CKDu was 18.6%.^40^ Importantly, these studies used only 1 laboratory result to define CKD. If we had used the same criteria without a subsequent clinical diagnosis, the overall prevalence of CKD stage 3 to 5 would have been 10.6% in our study cohort. However, such an approach could have misclassified acute kidney injury as CKD, introducing measurement bias to the results. For this reason and time constraint, we finally chose a stricter definition for our primary outcome, revealing Taiwan's low CKD diagnostic rate. Our findings underscore the nationwide issue of undiagnosed CKD and the need to raise public health and medical community concerns.

The low prevalence of CKD diagnosis among individuals with an eGFR below 60 could also be attributed to patient awareness and physician inertia. In the previous study by Wen *et al.* in 2008, it was demonstrated that there was a low awareness rate of 3.5% among all CKD patients.^38^ It is important to note that the denominator (CKD by eGFR equation and definition) and the numerator (self-awareness or being informed by medical staff) in their study were determined at the time of patient recruitment. In our current study, we utilized data from NAHE, and the diagnosis of CKD could be made in follow-up medical visits within 1 year after participants had their blood drawn. It is true that at the time of blood collection, patients may not have been aware of their CKD status, which could potentially affect their subsequent participation in CKD-related follow-up. However, on completing the health screening (patient’s second visit for the examination report of NAHE), it is expected that physicians would inform patients of their results and help arrange appropriate medical follow-up. Therefore, the low diagnostic rate of CKD observed in our study may not solely be attributed to patients' lack of awareness but also warrants an exploration of physician inertia in initiating follow-up for CKD.

In summary, although the low prevalence of CKD diagnosis among individuals with an eGFR below 60 may partially be explained by patients’ lack of awareness, it is crucial to consider physician inertia as a contributing factor. Further research is needed to better understand and address these issues to improve CKD detection and management.

The current definition of CKDu used for epidemiological studies generally excludes only cases with diabetes and hypertension, leaving other potential causes of “nondiabetic and normotensive CKD” to be clarified before the diagnosis of CKDu. In other words, many epidemiologic studies have limited power to distinguish between CKDu and “nondiabetic and normotensive CKD”, which was also the case for our first definition of CKDu (CKDu_1). Therefore, the association found in our study between farming status and CKDu should be interpreted with this limitation in mind. It is worth noting that although we additionally excluded proteinuria and diagnosis of glomerular diseases and congenital urinary diseases in an alternative definition of CKDu (CKDu_2), we might inadvertently exclude some cases with transient proteinuria because the health examinations do not require a urine sample in early morning. This resulted in a smaller number of cases in CKDu_2, potentially contributing to the nonsignificant findings observed.

### Generalizability

The representativeness of the study population is an important consideration for interpreting research findings. From 2012 to 2018, 30% of adults utilized NAHE, which was offered to those aged 40 and above in general.^11^ As a result, the study may represent middle-aged and older populations more. Furthermore, the characteristics of Taiwanese farmers may differ from those in global CDKu hotspots such as Central America or South Asia. Taiwanese farmers are mostly self-employed and tend to be older (average age 68) because of the migration of labor from rural to urban areas.^17^ These factors can impact the study findings’ generalizability to other farmers’ populations in different regions. To summarize, the unique characteristics of the study population need to be considered when applying these findings to other populations.

### Limitations

Several limitations should be considered when interpreting the results. First, the study was retrospectively observational, and we could not control for all potential confounders. We did not have exposure data on potential nephrotoxic agents, such as agrochemicals, heavy metals, silica, or the use of analgesics. This could lead to a biased estimate of the risk of CKD. In addition, we were unable to adjust for socioeconomic factors, such as educational level, because the database used in our study, the NAHE database, does not provide this information. Second, we were unable to subgroup agricultural workers based on the types of crops they cultivate and the influence of machinery on agricultural practices. The specific details of their working patterns, including the use of machinery, can significantly impact the associated heat strain. Furthermore, we used Farmer's Health Insurance status to determine participants’ occupations as farmers. Although it is a common practice in population-based studies utilizing databases such as NAHE, it might not be accurate. In our case, the use of Farmer's Health Insurance status to determine participants' occupation as farmers may slightly overestimate the proportion of farmers (13.4% in our study vs. 11.5% in 2015 Taiwan Agricultural Census).^29^ Third, there may have been selection bias in the recruitment of participants. Those who lacked health awareness or were too busy or sick may have not yet participated in the health screening program, which might underestimate the risk of CKD/CKDu. Fourth, the study relied on self-reported data, which could introduce recall bias. For example, participants may have underreported or overreported their occupational status and other lifestyle and clinical factors, leading to an overestimation or underestimation of the risk of CKD/CKDu. Finally, the study did not investigate potential mechanisms underlying the observed associations between outdoor heat exposure and kidney function decline. It is unclear whether these associations are causal or reflect shared risk factors and further research is needed to clarify these relationships. Therefore, caution should be exercised in generalizing our findings to other populations.

## Conclusion

Farmers in Taiwan are at higher risk of CKDu but not overall CKD compared to nonfarmers. Increased outdoor heat exposure was associated with the development of CKD, and middle-aged participants and those with diabetes are particularly vulnerable. The prevalence of CKD diagnosis was low among both farmers and nonfarmers, highlighting the need for improved detection and monitoring of the disease in Taiwan. The unique characteristics of the Taiwanese population, including the farmers’ older age, should also be considered when generalizing these findings to other populations. Further research is needed to better understand the complex interplay of risk factors contributing to CKD in agricultural communities.

## Disclosure

All the authors declared no competing interests.

## Supplementary Material

Supplementary File (PDF)

**Figure S1.** Subgroup analysis for the association between CKD and the average ambient temperature within 9 months before the examination.

**Table S1.** Service Criteria National Adult Health Examination (NAHE).

**Table S2.** ICD codes used in this study.

**Table S3.** Associations between CKD and different types of average ambient temperature and time lags.

**STROBE Statement.**
