# Supplementary material for: Chronic Kidney Disease Among Agricultural Workers in Taiwan: A Nationwide Population-Based Study
Source: Kidney Int Rep. 2023 Sep 9;8(12):2677–89. doi: 10.1016/j.ekir.2023.09.004 (PMC10719565; doi:10.1016/j.ekir.2023.09.004)
Supplement: Supplementary File (PDF) [file mmc1.pdf]

Supporting Information for

Chronic Kidney Disease of among Agricultural Workers in Taiwan: A Nationwide Population-Based Study

Authors: Che-Jui Chang [a,b], Hsiao-Yu Yang [a,c,d,e]

Affiliations:

[a] Institute of Occupational and Environmental Health Sciences, National Taiwan University College of Public Health, Taipei, Taiwan

[b] Department of Family Medicine, National Taiwan University Hospital Hsin-Chu Branch, Hsinchu, Taiwan

[c] Department of Public Health, National Taiwan University College of Public Health, Taipei, Taiwan

[d] Department of Environmental and Occupational Medicine, National Taiwan University Hospital, Taipei, Taiwan

[e] Population Health and Welfare Research Center, National Taiwan University College of Public Health, Taipei, Taiwan

Corresponding author: Dr. Hsiao-Yu Yang

Institute of Occupational and Environmental Health Sciences

National Taiwan University College of Public Health, Taipei, Taiwan

No. 17 Xuzhou Road, Taipei 100, Taiwan

Tel.: +886-2-3366-8102

hyang@ntu.edu.tw

Contents of this file

- STROBE Statement
- Table S1. Service Criteria National Adult Health Examination (NAHE)
- Table S2. ICD codes used in this study
- Table S3. Associations between CKD and different types of average ambient temperature and time lags
- Figure S1. Subgroup analysis for the association between CKD and the average ambient temperature within 9 months before the examination

STROBE Statement—checklist of items that should be included in reports of observational studies

	Item No	Recommendation	Page No
**Title and abstract**	1	(*a*) Indicate the study's design with a commonly used term in the title or the abstract	1
		(*b*) Provide in the abstract an informative and balanced summary of what was done and what was found	2
**Introduction**			
Background/rationale	2	Explain the scientific background and rationale for the investigation being reported	3
Objectives	3	State specific objectives, including any prespecified hypotheses	3
**Methods**			
Study design	4	Present key elements of study design early in the paper	4
Setting	5	Describe the setting, locations, and relevant dates, including periods of recruitment, exposure, follow-up, and data collection	4-6
Participants	6	(*a*) *Cohort study*—Give the eligibility criteria, and the sources and methods of selection of participants. Describe methods of follow-up<br>*Case-control study*—Give the eligibility criteria, and the sources and methods of case ascertainment and control selection. Give the rationale for the choice of cases and controls<br>*Cross-sectional study*—Give the eligibility criteria, and the sources and methods of selection of participants	4-5
		(*b*) *Cohort study*—For matched studies, give matching criteria and number of exposed and unexposed<br>*Case-control study*—For matched studies, give matching criteria and the number of controls per case	6
Variables	7	Clearly define all outcomes, exposures, predictors, potential confounders, and effect modifiers. Give diagnostic criteria, if applicable	4-6
Data sources/ measurement	8*	For each variable of interest, give sources of data and details of methods of assessment (measurement). Describe comparability of assessment methods if there is more than one group	4-6
Bias	9	Describe any efforts to address potential sources of bias	6-7
Study size	10	Explain how the study size was arrived at	NA
Quantitative variables	11	Explain how quantitative variables were handled in the analyses. If applicable, describe which groupings were chosen and why	6-7
Statistical methods	12	(*a*) Describe all statistical methods, including those used to control for confounding	6-7
		(*b*) Describe any methods used to examine subgroups and interactions	7
		(*c*) Explain how missing data were addressed	6, Table 1
		(*d*) *Cohort study*—If applicable, explain how loss to follow-up was addressed<br>*Case-control study*—If applicable, explain how matching of cases and controls was addressed<br>*Cross-sectional study*—If applicable, describe analytical methods taking account of sampling strategy	6
		(*e*) Describe any sensitivity analyses	7
**Results**			
Participants	13*	(a) Report numbers of individuals at each stage of study—eg numbers potentially eligible, examined for eligibility, confirmed eligible, included in the study, completing follow-up, and analysed	7, Fig. 1
		(b) Give reasons for non-participation at each stage	Fig. 1
		(c) Consider use of a flow diagram	Fig. 1
Descriptive data	14*	(a) Give characteristics of study participants (eg demographic, clinical, social) and information on exposures and potential confounders	7-8, Table. 1, Table. 2

		(b) Indicate number of participants with missing data for each variable of interest	Table. 1
		(c) *Cohort study*—Summarise follow-up time (eg, average and total amount)	4
Outcome data	15*	*Cohort study*—Report numbers of outcome events or summary measures over time	
		Case-control study—Report numbers in each exposure category, or summary measures of exposure	7-8, Table. 1
		Cross-sectional study—Report numbers of outcome events or summary measures	
Main results	16	(*a*) Give unadjusted estimates and, if applicable, confounder-adjusted estimates and their precision (eg, 95% confidence interval). Make clear which confounders were adjusted for and why they were included	8, Table. 3
		(*b*) Report category boundaries when continuous variables were categorized	Table. 3
		(*c*) If relevant, consider translating estimates of relative risk into absolute risk for a meaningful time period	NA
Other analyses	17	Report other analyses done—eg analyses of subgroups and interactions, and sensitivity analyses	8-9
**Discussion**			
Key results	18	Summarise key results with reference to study objectives	9
Limitations	19	Discuss limitations of the study, taking into account sources of potential bias or imprecision. Discuss both direction and magnitude of any potential bias	14
Interpretation	20	Give a cautious overall interpretation of results considering objectives, limitations, multiplicity of analyses, results from similar studies, and other relevant evidence	10-13
Generalisability	21	Discuss the generalisability (external validity) of the study results	13-14
**Other information**			
Funding	22	Give the source of funding and the role of the funders for the present study and, if applicable, for the original study on which the present article is based	16

*Give information separately for cases and controls in case-control studies and, if applicable, for exposed and unexposed groups in cohort and cross-sectional studies.

Table S1. Service Criteria National Adult Health Examination (NAHE)

Target Population	Frequency	Service Items
Age 40 and above but under 65	Once every 3 years	1. Basic Information: Questionnaire (medical history, family history, medication history, health behaviors, depression screening, etc.)<br><br>2. Physical Examination: General physical examination, height, weight, blood pressure, body mass index (BMI), waist circumference<br><br>3. Laboratory Tests:<br>(1) Urine Test: urine protein (qualitative or quantitative)<br>(2) Estimated Glomerular Filtration Rate (eGFR) calculation<br>(3) Blood Biochemical Tests: Aspartate aminotransferase (GOT), Alanine aminotransferase (GPT), Creatinine, Blood glucose, Blood lipids (Total cholesterol, Triglycerides, High-density lipoprotein cholesterol, Low-density lipoprotein cholesterol calculation).<br>(4) Hepatitis B Surface Antigen (HBsAg) and Hepatitis C Antibody (anti-HCV): Once in a lifetime for individuals aged 45 to 79. Once in a lifetime for individuals of indigenous ethnicity aged 40 to 79. (updated in 28 September 2020)<br><br>4. Health Consultation: Smoking cessation, alcohol moderation, betel nut cessation, regular exercise, maintaining a healthy weight, healthy diet, injury prevention, oral health.
Indigenous individuals aged 55 and above, individuals with poliomyelitis aged 35 and above, individuals aged 65 and above	Once a year	

Table S2. ICD codes used in this study

	Diagnosis	ICD-9-CM and ICD-10-CM	Additional requirements
Outcome of interest	CKD	585, 586, 587, 588, D631, E0822, E0922, E1022, E1122, E1322, I12, I13, N18	Number of outpatient visits ≥2 in one year or one hospitalization with the diagnosis; an eGFR < 60 mL/min/1.73 m².
	CKDu	585, 586, 587, 588, D631, N18	Number of outpatient visits ≥2 in one year or one hospitalization with the diagnosis; an eGFR < 60 mL/min/1.73 m²; exclusion of cases with hypertension or diabetes.
Comorbidities	Hypertension	401, 402, 405, I10, I11, I15	Number of outpatient visits ≥2 in one year or one hospitalization with the diagnosis
	Diabetes	250, E08, E09, E10, E11, E12, E13	Number of outpatient visits ≥2 in one year or one hospitalization with the diagnosis
	Hyperlipidemia	272, E78	Number of outpatient visits ≥2 in one year or one hospitalization with the diagnosis
	Heart disease	393, 394, 395, 396, 397, 398, 410, 427, 428, 429, I05, I06, I07, I08, I09, I20, I21, I22, I23, I24, I25, I46, I47, I48, I49, I50, I51, I52, I97, R001	Number of outpatient visits ≥2 in one year or one hospitalization with the diagnosis
	Glomerular disease	580, 581, 582, 583, N00, N01, N02, N03, N04, N05, N06, N07, N08	Number of outpatient visits ≥2 in one year or one hospitalization with the diagnosis
	Congenital urinary disease	753, Q60, Q61, Q62, Q63, Q64	Number of outpatient visits ≥2 in one year or one hospitalization with the diagnosis
	Chronic liver disease	5710, 5711, 5713, 5714, 5717, 5718, 5719, K700, K701, K709, K73, K754, K758, K759, K760, K7689, K769	Number of outpatient visits ≥2 in one year or one hospitalization with the diagnosis
	Gout / hyperuricemia	274, M10, E79	Number of outpatient visits ≥2 in one year or one hospitalization with the diagnosis
	Urolithiasis	5920, 5921, 5929, 5940, 5941, 5942, 5948, 5949, 7880, V1301, N132, N200, N201, N202, N209, N210, N211, N218, N219	One outpatient visit, one emergency department visit, or one hospitalization with the diagnosis

Abbreviation: CKD, chronic kidney disease; CKDu, chronic kidney disease of undetermined etiology; eGFR, estimated glomerular filtration rate; ICD, International Statistical Classification of Diseases and Related Health Problems.

Table S3. Associations between CKD and different types of average ambient temperature and time lags

Lag structure	Daily mean temperature	Daily maximum temperature	Daily minimum temperature	Daily mean WBGT	Daily maximum WBGT	Daily minimum WBGT
	**Adjusted odds ratio of CKD for different types of average ambient temperatures**					
**Single lag: average ambient temperature of the Nth month before the examination (°C)**						
lag 1	**1.005 (1.003–1.008)**<br>$p < 0.001$	**1.006 (1.003–1.008)**<br>$p < 0.001$	**1.004 (1.001–1.006)**<br>$p = 0.002$	**1.005 (1.003–1.007)**<br>$p < 0.001$	**1.006 (1.003–1.008)**<br>$p < 0.001$	**1.006 (1.003–1.008)**<br>$p < 0.001$
lag 2	1.002 (0.999–1.004)<br>$p = 0.117$	**1.003 (1.000–1.005)**<br>$p = 0.018$	1.000 (0.998–1.003)<br>$p = 1.000$	1.002 (0.999–1.004)<br>$p = 0.117$	**1.003 (1.001–1.006)**<br>$p = 0.018$	**1.003 (1.001–1.006)**<br>$p = 0.018$
lag 3	0.998 (0.995–1.000)<br>$p = 0.117$	0.999 (0.996–1.001)<br>$p = 0.442$	0.996 (0.994–0.999)<br>$p = 0.002$	0.998 (0.995–1.000)<br>$p = 0.117$	0.999 (0.996–1.001)<br>$p = 0.442$	0.999 (0.996–1.001)<br>$p = 0.442$
lag 4	0.995 (0.993–0.998)<br>$p < 0.001$	0.997 (0.994–0.999)<br>$p = 0.019$	0.994 (0.992–0.997)<br>$p < 0.001$	0.995 (0.993–0.998)<br>$p < 0.001$	0.997 (0.994–0.999)<br>$p = 0.019$	0.997 (0.994–0.999)<br>$p = 0.019$
lag 5	0.995 (0.993–0.998)<br>$p < 0.001$	0.996 (0.993–0.998)<br>$p = 0.002$	0.995 (0.992–0.997)<br>$p < 0.001$	0.995 (0.993–0.998)<br>$p < 0.001$	0.996 (0.993–0.998)<br>$p = 0.002$	0.996 (0.993–0.998)<br>$p = 0.002$
lag 6	0.997 (0.995–1.000)<br>$p = 0.019$	0.998 (0.995–1.000)<br>$p = 0.117$	0.997 (0.995–1.000)<br>$p = 0.019$	0.997 (0.995–1.000)<br>$p = 0.019$	0.998 (0.995–1.000)<br>$p = 0.117$	0.998 (0.995–1.000)<br>$p = 0.117$
lag 7	1.002 (1.000–1.005)<br>$p = 0.116$	1.002 (1.000–1.005)<br>$p = 0.116$	1.002 (0.999–1.004)<br>$p = 0.117$	1.002 (0.999–1.004)<br>$p = 0.117$	1.002 (1.000–1.005)<br>$p = 0.116$	1.002 (1.000–1.005)<br>$p = 0.116$
lag 8	**1.007 (1.004–1.009)**<br>$p < 0.001$	**1.007 (1.004–1.010)**<br>$p < 0.001$	**1.007 (1.004–1.009)**<br>$p < 0.001$	**1.007 (1.004–1.009)**<br>$p < 0.001$	**1.007 (1.004–1.010)**<br>$p < 0.001$	**1.007 (1.004–1.010)**<br>$p < 0.001$
lag 9	**1.012 (1.009–1.015)**<br>$p < 0.001$	**1.012 (1.010–1.015)**<br>$p < 0.001$	**1.011 (1.009–1.014)**<br>$p < 0.001$	**1.012 (1.009–1.015)**<br>$p < 0.001$	**1.013 (1.010–1.015)**<br>$p < 0.001$	**1.013 (1.010–1.015)**<br>$p < 0.001$
lag 10	**1.015 (1.012–1.018)**<br>$p < 0.001$	**1.016 (1.013–1.018)**<br>$p < 0.001$	**1.014 (1.012–1.017)**<br>$p < 0.001$	**1.015 (1.012–1.018)**<br>$p < 0.001$	**1.016 (1.013–1.019)**<br>$p < 0.001$	**1.016 (1.013–1.019)**<br>$p < 0.001$
lag 11	**1.014 (1.011–1.017)**<br>$p < 0.001$	**1.015 (1.012–1.017)**<br>$p < 0.001$	**1.013 (1.011–1.016)**<br>$p < 0.001$	**1.014 (1.012–1.017)**<br>$p < 0.001$	**1.015 (1.013–1.018)**<br>$p < 0.001$	**1.015 (1.013–1.018)**<br>$p < 0.001$
lag 12	**1.011 (1.008–1.013)**<br>$p < 0.001$	**1.011 (1.009–1.014)**<br>$p < 0.001$	**1.010 (1.007–1.012)**<br>$p < 0.001$	**1.011 (1.008–1.013)**<br>$p < 0.001$	**1.012 (1.009–1.014)**<br>$p < 0.001$	**1.012 (1.009–1.014)**<br>$p < 0.001$
**Cumulative lag: average ambient temperature within N months before the examination (°C)**						
lag 0-1	**1.005 (1.003–1.008)**<br>$p < 0.001$	**1.006 (1.003–1.008)**<br>$p < 0.001$	**1.004 (1.001–1.006)**<br>$p = 0.002$	**1.005 (1.003–1.007)**<br>$p < 0.001$	**1.006 (1.003–1.008)**<br>$p < 0.001$	**1.004 (1.002–1.006)**<br>$p < 0.001$
lag 0-2	**1.004 (1.001–1.006)**<br>$p = 0.002$	**1.005 (1.002–1.007)**<br>$p < 0.001$	1.002 (1.000–1.005)<br>$p = 0.116$	**1.004 (1.001–1.006)**<br>$p = 0.002$	**1.005 (1.002–1.007)**<br>$p < 0.001$	**1.003 (1.000–1.005)**<br>$p = 0.018$

lag 0-3	1.002 (0.999–1.005)<br>$p = 0.192$	**1.003 (1.000–1.006)**<br>$p = 0.049$	1.000 (0.998–1.003)<br>$p = 1.000$	1.002 (0.999–1.004)<br>$p = 0.117$	**1.003 (1.000–1.006)**<br>$p = 0.049$	1.001 (0.998–1.003)<br>$p = 0.441$
lag 0-4	1.000 (0.997–1.003)<br>$p = 1.000$	1.001 (0.998–1.004)<br>$p = 0.524$	0.998 (0.995–1.001)<br>$p = 0.193$	1.000 (0.997–1.003)<br>$p = 1.000$	1.002 (0.999–1.005)<br>$p = 0.192$	0.999 (0.996–1.001)<br>$p = 0.442$
lag 0-5	0.998 (0.995–1.002)<br>$p = 0.266$	1.000 (0.997–1.003)<br>$p = 1.000$	0.997 (0.993–1.000)<br>$p = 0.093$	0.998 (0.995–1.002)<br>$p = 0.266$	1.000 (0.997–1.003)<br>$p = 1.000$	0.997 (0.994–1.000)<br>$p = 0.050$
lag 0-6	0.997 (0.994–1.001)<br>$p = 0.093$	0.999 (0.996–1.003)<br>$p = 0.587$	0.995 (0.992–0.999)<br>$p = 0.005$	0.997 (0.994–1.001)<br>$p = 0.093$	0.999 (0.996–1.003)<br>$p = 0.587$	0.995 (0.992–0.999)<br>$p = 0.005$
lag 0-7	0.998 (0.994–1.002)<br>$p = 0.333$	1.000 (0.996–1.004)<br>$p = 1.000$	0.996 (0.992–1.000)<br>$p = 0.050$	0.998 (0.994–1.002)<br>$p = 0.333$	1.000 (0.996–1.004)<br>$p = 1.000$	0.995 (0.991–0.999)<br>$p = 0.014$
lag 0-8	1.000 (0.996–1.005)<br>$p = 1.000$	1.003 (0.998–1.007)<br>$p = 0.192$	0.998 (0.993–1.002)<br>$p = 0.391$	1.000 (0.995–1.005)<br>$p = 1.000$	1.003 (0.998–1.007)<br>$p = 0.192$	0.997 (0.993–1.002)<br>$p = 0.193$
lag 0-9	**1.005 (1.000–1.010)**<br>$p = 0.049$	**1.007 (1.002–1.013)**<br>$p = 0.012$	1.002 (0.997–1.007)<br>$p = 0.441$	**1.005 (1.000–1.010)**<br>$p = 0.049$	**1.008 (1.003–1.013)**<br>$p = 0.002$	1.002 (0.997–1.007)<br>$p = 0.441$
lag 0-10	**1.012 (1.006–1.017)**<br>$p < 0.001$	**1.014 (1.009–1.020)**<br>$p < 0.001$	**1.008 (1.003–1.014)**<br>$p = 0.004$	**1.012 (1.006–1.018)**<br>$p < 0.001$	**1.015 (1.010–1.021)**<br>$p < 0.001$	**1.009 (1.003–1.015)**<br>$p = 0.003$
lag 0-11	**1.019 (1.013–1.025)**<br>$p < 0.001$	**1.021 (1.015–1.027)**<br>$p < 0.001$	**1.015 (1.009–1.021)**<br>$p < 0.001$	**1.020 (1.014–1.026)**<br>$p < 0.001$	**1.023 (1.016–1.029)**<br>$p < 0.001$	**1.018 (1.011–1.024)**<br>$p < 0.001$
lag 0-12	**1.023 (1.017–1.029)**<br>$p < 0.001$	**1.025 (1.019–1.031)**<br>$p < 0.001$	**1.019 (1.013–1.025)**<br>$p < 0.001$	**1.025 (1.018–1.031)**<br>$p < 0.001$	**1.027 (1.021–1.033)**<br>$p < 0.001$	**1.023 (1.017–1.030)**<br>$p < 0.001$

The table presents the odds ratios and corresponding 95% confidence intervals of chronic kidney disease (CKD) per unit increase in different types of average ambient temperature (°C) and lag structures. In this study, the default ambient temperature was calculated as the arithmetic average of the daily mean temperature. The hourly outdoor wet bulb globe temperature (WBGT) was estimated using the Liljegren formula, implemented in the R package "HeatStress" (available at https://github.com/anacv/HeatStress).
The lag structures included single-month lags and cumulative-month lags. For instance, lag 3 represents the average ambient temperature of the third month before the health examination, while lag 0-12 represents the average ambient temperature within the 12 months preceding the examination.
The odds ratios of CKD were adjusted for factors such as age, sex, occupation, residential region, lifestyle factors, body mass index (BMI), and comorbidities.

Figure S1. Subgroup analysis for the association between CKD and the average ambient temperature within 9 months before the examination

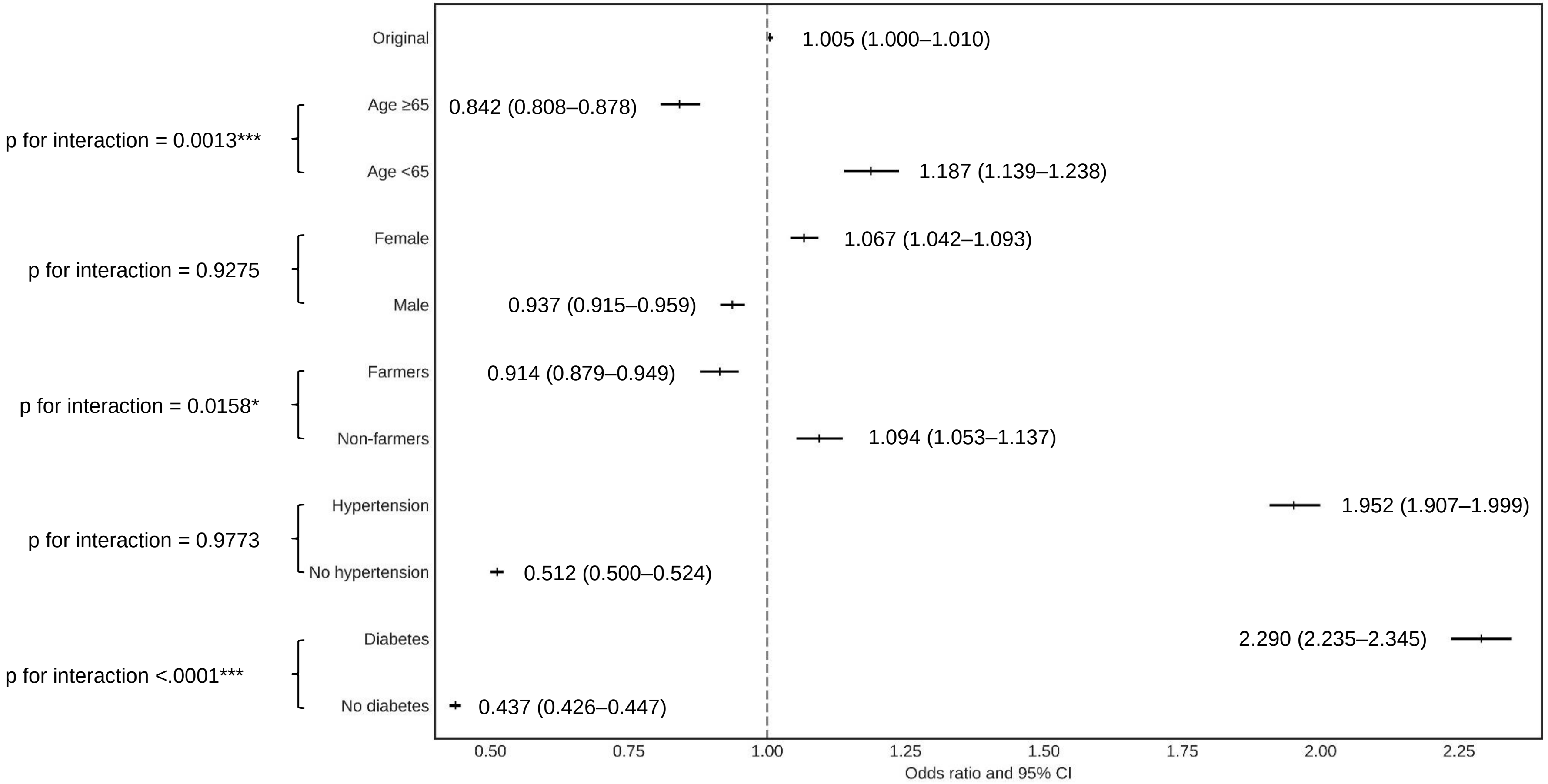

Legend: For the original study population and various subgroups, the error bar plot presents the odds ratios and corresponding 95% confidence intervals of chronic kidney disease (CKD) per unit increase in average ambient temperature (°C) within the 9 months preceding the examination. The odds ratios were adjusted for factors including age, sex, occupation, residential region, lifestyle factors, body mass index (BMI), and comorbidities. In the plot, the vertical bars represent the odds ratios, while the solid horizontal lines represent the corresponding 95% confidence intervals. The vertical dashed line represents an odds ratio of 1, indicating no increased risk. Hence, any vertical bar with its horizontal line positioned entirely to the right of this dashed line indicates an elevated risk of CKD associated with increased temperature.
